# Serum chemokines combined with multi-modal imaging to evaluate atherosclerotic plaque stability in patients undergoing carotid endarterectomy

**DOI:** 10.3389/fneur.2025.1537161

**Published:** 2025-03-31

**Authors:** Xiaofan Yuan, Lei Guo, Hong Chen, Yang Gao, Fuqiang Guo, Jie Huang, Chuan Jiang, Zhenyu Wang

**Affiliations:** ^1^Sichuan Provincial People's Hospital, School of Medicine, University of Electronic Science and Technology of China, Chengdu, China; ^2^Xindu District People's Hospital of Chengdu, Chengdu, China; ^3^The Affiliated Hospital, Southwest Medical University, Luzhou, China

**Keywords:** carotid artery stenosis, CEA, multi-imaging, atherosclerosis, chemokine

## Abstract

**Background:**

Although imaging tools are crucial in identifying features of atherosclerotic plaque, there remains a lack of consensus on the use of serological markers for assessing high-risk plaques.

**Methods:**

Patients diagnosed with CAS who met the criteria for CEA were categorized as the operation group, while those without CAS were designated as the control group. Multi-modal imaging was conducted pre- and post-CEA to evaluate plaque features, such as the volume of calcification and LRNC, intra-plaque hemorrhage, and the degree of carotid stenosis. Serum chemokine levels were measured in both groups before CEA and on the 7th day post-surgery. Morphological features of carotid artery specimens were assessed using H&E and IHC (CD68 and *α*-SMA) staining to evaluate plaque stability.

**Results:**

No significant differences in the degree of CAS between the operation and control groups. Among the operation group, 26 out of 52 patients were identified as vulnerable plaques. The volume of LRNC was significantly higher in vulnerable plaque, whereas the volume of calcification was significantly lower in vulnerable plaque compared to stable plaque confirmed by multi-modal imaging. Vulnerable plaque exhibited a thin fibrous cap covered an LRNC, intra-plaque hemorrhage, and macrophage infiltration. Stable plaque were characterized by small lipid cores covered by a thick fibrous cap, with minimal macrophage infiltration. Chemokine levels were significantly elevated in CAS patients compared to controls, and decreased significantly on the 7th day post-CEA. In patients with vulnerable plaque, lower levels of CX3CL1, CXCL12, CCL19, and CCL21, but higher levels of CCL2 and CCL5, were observed compared to patients with stable plaque. Correlation analysis further indicated that CX3CL1 and CXCL12 levels were positively associated with calcification volume. While CCL2 and CCL5 levels were positively associated, and CCL19 and CCL21 negatively associated, with LRNC volume. Multivariate analysis suggested that CXCL12 was an independent protective factor and LRNC volume as an independent risk factor for plaque vulnerability. The combination with multi-modal imaging and serological markers enhanced both the sensitivity (87.31%) and specificity (92.31%) in predicting plaque stability, with an AUC of 0.9001.

**Conclusion:**

Combining multi-modal imaging with serological markers provides a more comprehensive evaluation of atherosclerotic plaque features.

## 1 Background

Stroke is the most prevalent cause for hospital admission in neurology and has garnered significant attention due to its high rates of incidence, mortality, and disability. Ischemic stroke, in particular, accounts for approximately 87% of all stroke cases and remains a leading cause of death among urban populations worldwide (1, 2). Furthermore, the incidence of stroke continues to rise annually, imposing a substantial burden on both families and healthcare systems (3). Previous studies (4–8) have underscored that effective prevention and treatment of ischemic stroke should prioritize interventions aimed at stabilizing vulnerable atherosclerotic plaque, rather than focusing solely on alleviating carotid artery stenosis (CAS). Promoting plaque stabilization and regression is considered more efficacious for preventing ischemic events. Nevertheless, the factors, underlying mechanisms, and optimal interventions influencing plaque stability remain poorly understood. Thus, advancing the concept of vulnerable atherosclerotic plaque offers significant scientific value for the early identification of high-risk plaque and for mitigating the risk of ischemic stroke.

Current imaging modalities for assessing CAS include carotid ultrasound, Computed Tomography Angiography (CTA), and Magnetic Resonance Angiography (MRA), each with distinct advantages and limitations. Carotid ultrasound, characterized by ease of use, reproducibility, and low cost, is one of the most widely used diagnostic tools in clinical practice (9, 10). However, its effectiveness is constrained in cases involving high carotid bifurcation or a short neck, and severe calcification can also interfere with intra-plaque visualization (11). CTA is highly effective in quantifying the degree of lumen stenosis and is particularly sensitive in detecting plaque calcification, but it demonstrates relatively poor accuracy in differentiating lipid cores from hemorrhages within plaque (12, 13). Although MRA can effectively identify specific components of atherosclerotic plaque, including the fibrous cap, lipid-rich necrotic core (LRNC), intra-plaque hemorrhage, and neovascularization, it is associated with several limitations, such as long acquisition times, sensitivity to patient motion, high costs, and limited sensitivity to calcified components (14–16). Given the limitations of these individual imaging tools in assessing the full spectrum of plaque features, we propose that integrating imaging techniques with serological markers may enhance the evaluation of plaque stability, thereby improving preoperative planning and the accuracy of stroke risk prediction.

Chemokines and their receptors are widely expressed across endothelial cells, smooth muscle cells (SMCs), monocytes, and macrophages within the cardio- and cerebrovascular systems. Numerous studies (17–33) have established that chemokines participate in crucial processes underlying the development, progression, and regression of atherosclerosis. In atherosclerotic plaque, the expression of Chemokine Ligand 2 (CCL2) is significantly elevated compared to normal arterial tissue (18). The CCL2-Chemokine Receptor 2 (CCR2) axis facilitates the recruitment of circulating monocytes into atherosclerotic plaque, where these monocytes differentiate into macrophages. The macrophages subsequently proliferate, ingest oxidized low-density lipoproteins (ox-LDL) to form foam cells, and coordinate inflammatory responses, thereby promoting atherogenesis (19–21). Depletion of CCL2 or selective deletion of CCR2 significantly attenuates atherosclerotic lesions in low-density lipoprotein receptor-deficient (LDLR−/−) mice (22) and Apolipoprotein E knockout (ApoE −/−) mice (23) by inhibiting macrophage accumulation within plaque. Furthermore, platelets provide chemokines that localize monocytes to atherosclerotic sites, facilitating adhesion and migration, thus contributing to atherogenesis (24). Notably, chemokines such as CCL5 and C-X-C Motif Ligand 4 (CXCL4), which are abundant in platelets, attract monocytes expressing Chemokine Receptor 5 (CCR5) and Chemokine Receptor 1 (CCR1) (25). Disruption of CCR5 expression or inhibition of CCR5 function via antagonists results in a marked reduction in macrophage infiltration and lesion size (26, 27). During the progression of atherosclerosis, the continuous accumulation of ox-LDL and debris within subendothelial macrophages ultimately leads to macrophage apoptosis. Conversely, studies (28, 29) have found that the C-X3-C Motif Receptor 1 (CX3CR1)/C-X3-C Motif Ligand 1 (CX3CL1) axis can attenuate lipid-induced macrophage apoptosis, thereby enhancing cellular survival and delaying plaque regression. CX3CL1 has also been implicated in the osteogenic transformation of SMCs, contributing to plaque calcification (30). Akhtar et al. (31) reported that mice injected with C-X-C Motif Ligand 12 (CXCL12) exhibited thicker fibrous caps compared to control groups, suggesting that CXCL12 may facilitate the accumulation of smooth muscle progenitor cells within plaque, thereby enhancing plaque stability. In contrast, plaque regression is largely mediated by the emigration of foam cells and macrophages from the lipid core. Chemokine Ligands 19 (CCL19) and 21 (CCL21), after binding to Chemokine Receptor 7 (CCR7), have been shown to promote macrophage emigration from plaque, thereby reducing plaque burden (32–34). Although chemokines have demonstrated vital roles in atherosclerosis, clinical trials targeting chemokine antagonists or receptors have yielded disappointing results. Failures have been attributed to factors such as off-target effects, cross-reactivity among structurally similar receptors, and discrepancies between animal models and human disease. The redundancy of chemokines in regulating immune cell homeostasis, recruitment, and activation may also complicate effective targeting (35). Given the crucial role of the aforementioned chemokines in the development and progression of atherosclerosis, there remains a lack of research on their expression in serum and their relationship with plaque stability. We conducted an in-depth analysis of the expression, dynamic changes, and correlation of these chemokines with plaque stability in serum.

Therefore, in this study, we sought to combine multi-modal imaging with morphological analyses to explore the correlation between plaque stability and serum chemokine concentrations in patients before and after carotid endarterectomy (CEA). This integrated approach aims to establish a foundation for better controlling atherosclerosis progression and preventing stroke.

## 2 Materials and methods

### 2.1 Study subjects

This study included patients diagnosed with CAS or occlusion through imaging tools (carotid ultrasound and CTA) who met the criteria for CEA (36) and were willing to undergo CEA at the Department of Neurosurgery from January 2023 to August 2024. These patients constituted the operation group. Patients without CAS, as determined by carotid ultrasound and CTA, were included in the control group. Ethical approval was obtained from the Ethics Committee of Sichuan Provincial People’s Hospital, School of Medicine, University of Electronic Science and Technology of China, and all study participants provided written informed consents.

### 2.2 Inclusion and exclusion criteria

#### 2.2.1 Inclusion criteria

(i) Patients diagnosed with CAS or occlusion based on carotid ultrasound and CTA; (ii) Patients meeting the criteria for CEA (36) and willing to undergo surgery; (iii) Age between 18 and 80 years; (iv) Patients who provided written informed consent.

#### 2.2.2 Exclusion criteria

(i) Patients diagnosed with CAS or occlusion due to developmental disorders, inflammation, Moyamoya disease, or autoimmune-related conditions; (ii) Patients with previous CEA or endovascular therapy, complicated by subsequent ipsilateral lumen restenosis; (iii) Patients with malignant tumors of the head and neck; (iv) Patients deemed unsuitable for surgery.

### 2.3 CEA procedure

Under general anesthesia, patients were positioned with the neck extended and the head turned contralaterally to ensure optimal surgical exposure. A vertical incision was made along the anterior border of the sternocleidomastoid muscle, and tissue was dissected downwards to expose the carotid sheath. Following incision of the carotid sheath, the common carotid artery (CCA), external carotid artery (ECA), internal carotid artery (ICA), and superior thyroid artery were identified and temporarily occluded using vascular clamps (FB471R, Germany) to halt antegrade flow. The CCA and ICA were then incised longitudinally, and the atherosclerotic plaque was meticulously excised. The vascular lumen was irrigated with heparinized saline, followed by closure using a 6–0 proline non-absorbable suture (W8003T, US). After intraoperative angiographic confirmation of restored blood flow, the clamps were released, and the incision was closed in layers. Postoperatively, patients were treated with Aspirin (Bayer, Germany) 100 mg daily from one day before CEA and continued for six months thereafter.

### 2.4 Patient characteristics

Baseline demographic and clinical characteristics, including age, sex, height, weight, diabetes status, blood pressure, smoking, alcohol use, statin use, and history of stroke and transient ischemic attack (TIA) were documented. Serum parameters, including uric acid, creatinine, glycosylated hemoglobin (HbA1c), total cholesterol (TC), triglycerides (TG), high-density lipoprotein (HDL), and low-density lipoprotein (LDL), were also recorded.

## 3 Blood samples and CEA specimen analysis

### 3.1 Blood sample collection

On admission, 3 mL of fasting venous blood was collected from all patients in both the operation and control groups. In the operation group, additional blood samples were collected on the 7th day post-CEA. These samples were centrifuged at 2000 rpm for 20 min, and serum aliquots were stored at −80°C for subsequent analysis. Serum concentrations of CCL19 (AB-B23275, Abmart), CCL21 (AB-B22105, Abmart), CCL2 (AB-B29436, Abmart), CCL5 (AB-B217811, Abmart), CXCL12 (AB-B221059, Abmart), and CX3CL1 (AB-B24257, Abmart) were quantified using Enzyme-Linked Immunosorbent Assay (ELISA) kits, following the manufacturer’s protocols. Each sample was analyzed in triplicate, and the results were averaged for statistical purposes.

### 3.2 CEA specimen analysis

CEA specimens obtained from surgical patients were fixed in paraformaldehyde, embedded in paraffin, sectioned, and subsequently stained with hematoxylin and eosin (H&E) and immunohistochemistry (IHC) targeting Cluster of Differentiation 68 (CD68, 28,058-1-AP, Proteintech) and *α*-Smooth Muscle Actin (α-SMA, 14395-1-AP, Proteintech) antibodies. The staining procedures were performed as described previously (37, 38). CD68-positive cells were used as markers for macrophage infiltration following ox-LDL phagocytosis, while *α*-SMA-positive cells were indicative of SMCs involved in fibrous cap formation. According to established criteria (39), plaque were classified as vulnerable or stable based on morphological assessment of stained sections. Plaque were considered vulnerable if they met one major criterion or two minor criteria. Major Criteria: (i) Active inflammation (monocytes/macrophages or T-cell infiltration), (ii) Thin fibrous cap with LRNC, (iii) Endothelial denudation with platelet aggregation, (iv) Plaque fissuring, (v) Stenosis ≥90%. Minor Criteria: (i) Superficial calcified nodule, (ii) Glistening yellow appearance, (iii) Intraplaque hemorrhage, (iv) Endothelial dysfunction, (v) Positive (outward) remodeling.

## 4 Multi-modal imaging acquisition and post-processing

### 4.1 Carotid ultrasound and angiography

Prior to and on the 7th day following CEA, patients in the operation group underwent carotid ultrasound and angiography, as well as CTA and Computed Tomography Perfusion (CTP). The control group underwent carotid ultrasound, CTA, and CTP upon admission. For ultrasound and angiography, 5 mL of contrast agent (SonoVue, Bracco Suisse SA) was injected into the median cubital vein, followed by horizontal and longitudinal scans using the iE33 ultrasound system (Philips) and angiography unit (SC2000, Siemens). The bilateral CCA, ICA, and ECA were visualized from the root of the left aortic arch to the bifurcation of the right innominate artery. Parameters such as intima-media thickness, inner diameters, degree of stenosis, and average blood flow velocity were recorded. In patients with multiple plaque or stenoses, the most severe lesion was selected for analysis.

### 4.2 CT imaging

#### 4.2.1 CTA

CTA was conducted using a 256-slice CT scanner (SOMATOM Force, Germany), covering the area from the aortic arch to the C1 vertebra. CT parameters included section thickness of 5 mm, section interval of 5 mm, reconstruction interval of 0.6 mm, FOV of 20 cm, pitch of 0.5, and acquisition settings of 70 kVp/100 mA. CT images were independently evaluated by two radiologists, with discrepancies resolved by a third radiologist. CAS severity was assessed according to the North American Symptomatic Carotid Endarterectomy Trial standards (40, 41). Carotid plaque features were classified as follows: calcified plaque (attenuation >130 HU), soft plaque (attenuation 40–50 HU, indicative of hemorrhage or LRNC), and plaque ulceration (extension of contrast beyond the vascular lumen by ≥1 mm) (42, 43). Plaque volumes were measured using threshold segmentation with 3D Slicer software (National Institutes of Health, US) within a 2 cm region on either side of the carotid bifurcation.

#### 4.2.2 CTP

CTP images were uploaded to workstation for perfusion analysis. Regions of interest (ROIs) were defined bilaterally in the anterior and middle cerebral artery territories, and perfusion parameters, relative cerebral blood volume (rCBV), relative cerebral blood flow (rCBF), relative mean transit time (rMTT), and relative time to peak (rTTP) were calculated.

## 5 Statistical analysis

All statistical analyses were conducted using SPSS (IBM, version 28.0), and data visualization was performed using GraphPad Prism (GraphPad Software, version 10.3.0). Continuous variables with normal distribution were expressed as mean ± standard deviation, while skewed distributions were reported as median and interquartile range. Categorical variables were presented as frequencies and percentages. Comparisons of serum chemokine concentrations, hemodynamic parameters, and baseline characteristics were performed using paired t-tests or Analysis of Variance (‌‌ANOVA), contingent upon homogeneity of variance assumptions. When the assumption of homogeneity was violated, the Wilcoxon rank-sum test was applied. Correlations between serum chemokine levels and atherosclerotic plaque characteristics were analyzed using Spearman’s rank correlation. Univariate and multivariate logistic regression analyses were conducted to identify factors affecting plaque stability, with predictive accuracy evaluated by Receiver Operating Characteristics (ROC) curve analysis and under the curve (AUC). A *p*-value <0.05 was considered statistically significant.

## 6 Results

### 6.1 Baseline characteristics of patients

A total of 104 patients were enrolled in the study, comprising 52 patients who underwent CEA due to the presence of CAS, and 52 patients in the control group without CAS as confirmed by imaging assessments. The proportion of males in the operation and control groups was 61.53 and 53.85%, respectively. The mean age was 60.32 ± 9.20 years in the operation group and 58.68 ± 11.02 years in the control group. There were no statistically significant differences in baseline characteristics, including age, sex, Body Mass Index (BMI), alcohol consumption, diabetes status, smoking status, hypertension, blood lipid profiles, uric acid, and creatinine levels between the control and operation groups (Table 1). The statin use and history of ischemic cerebrovascular diseases had statistically significant differences between the two groups. Therefore, to investigate the potential influences of statin use and ischemic cerebrovascular disease history on the chemokines pattern and imaging parameters, we subsequently conducted detailed subgroup analysis.

**Table 1:** Baseline characteristics of study patients.

Variables	Control group	Operation group	*P*
Age (year)	60.32 ± 9.20	58.68 ± 11.02	0.95
Sex (male)	32/61.53%	28/53.85%	0.67
BMI (kg/m^2^)	22.71 ± 3.86	23.49 ± 4.22	0.71
Alcohol use	14/26.92%	12/23.08%	0.27
Diabetes	16/30.77%	14/26.92%	0.32
Current smoker	11/21.15%	9/17.31%	0.49
Former smoker	24/46.15%	25/48.08%	0.92
Hypertension	39/75%	34/65.38%	0.85
HbA1c (%)	6.34 ± 3.12	6.18 ± 3.17	0.97
TG (mmol/L)	4.06 ± 1.91	3.77 ± 2.04	0.42
TC (mmol/L)	1.38 ± 0.46	1.30 ± 0.53	0.49
LDL (mmol/L)	2.62 ± 1.09	2.31 ± 1.06	0.21
HDL (mmol/L)	0.98 ± 0.41	1.02 ± 0.55	0.65
Uric Acid (umol/L)	396.82 ± 102.39	402.29 ± 93.83	0.17
Creatinine (umol/L)	72.43 ± 30.13	80.20 ± 21.45	0.14
Statin use	15/28.84%	41/78.85%	< 0.01**
Stroke/TIA	3/5.77%	21/40.38%	< 0.01**

**P < 0.01, indicates statistical significance.

### 6.2 Multi-modal imaging changes pre- and post-CEA

**Figure 1:**
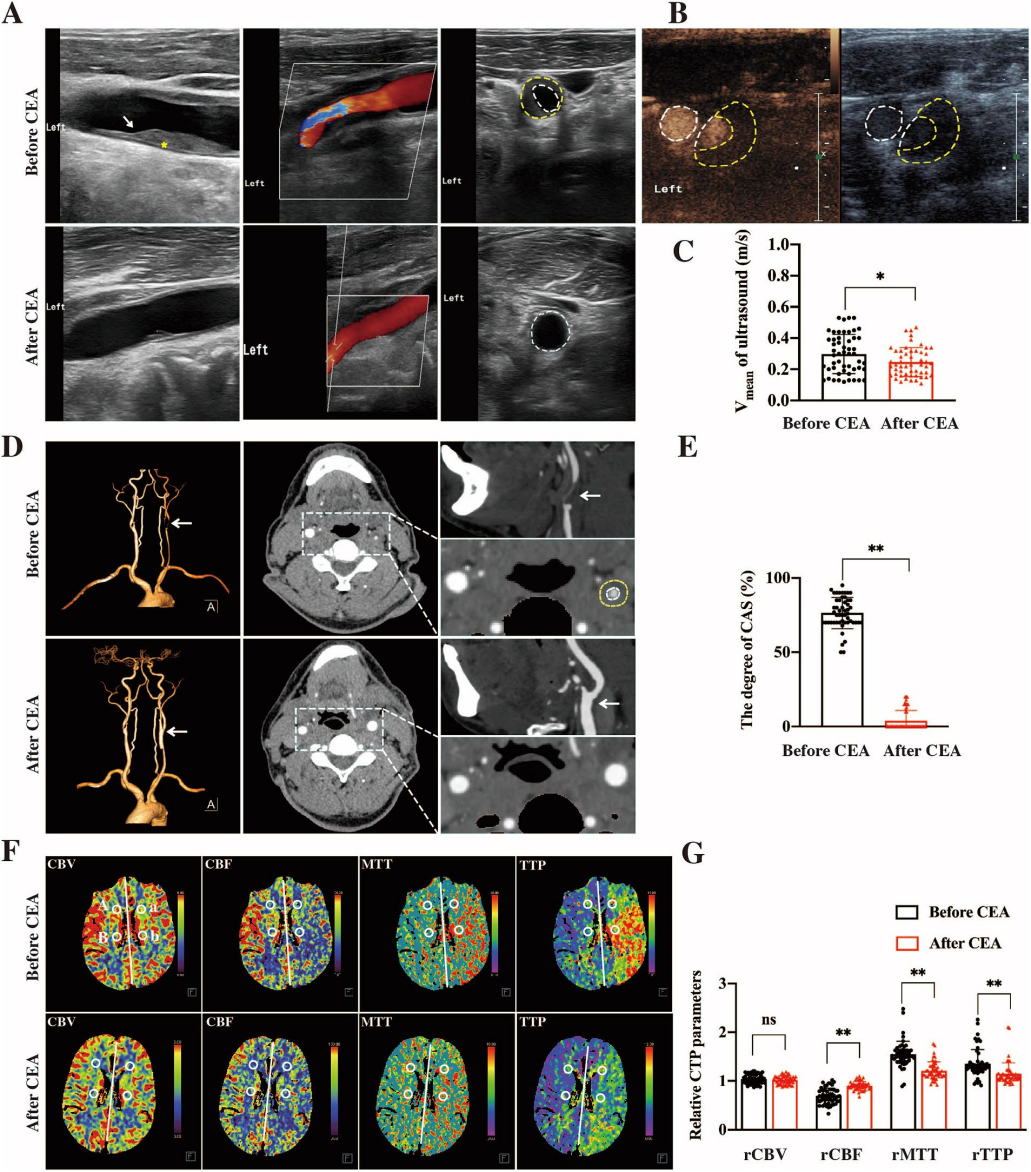
Multi-modal imaging changes pre- and post-CEA. In pre-CEA patients, vascular ultrasound identified a low and weak echogenic signal (indicated by the yellow * and white arrow) at the carotid bifurcation, which in an indicative of significant luminal stenosis **(A)**. Ultrasound angiography further demonstrated a filling defect of the contrast agent and accelerated intracavitary blood flow **(B)**. Following CEA in patients with CAS, the degree of stenosis was notably reduced, with restoration of normal blood flow dynamics. The average blood flow velocity significantly decreased in post-CEA patients compared to pre-CEA patients (**C**, *p < 0.05). CTA imaging demonstrated severe stenosis (> 90%) in the left CCA of pre-CEA patients, whereas significant improvement in stenosis was observed in post-CEA patients (**D**, white arrow). And post-CEA patients experienced a significant improvement in the degree of carotid stenosis (**E**, **p < 0.01). CTP imaging revealed that reduced cerebral perfusion on the ipsilateral side compared to the contralateral side of carotid stenosis **(F)**, which is characterized by prolonged rMTT and rTTP, decreased rCBF, and unaffected rCBV. And the hypoperfusion in the ipsilateral hemisphere after CEA gradually improved (**G**, **p < 0.01; ns indicates no statistical difference).

Prior to CEA, carotid ultrasound revealed a low and weak echo at the carotid bifurcation (Figure 1A). Ultrasound angiography demonstrated a contrast agent filling defect, with accelerated blood flow in the affected lumen (Figure 1B). These findings were consistent with the presence of atherosclerotic plaque causing luminal stenosis or occlusion. Post-CEA, ultrasound imaging showed a significant improvement, with stenosis relieved and forward blood flow restored. The average blood flow velocity significantly decreased from 0.30 ± 0.13 m/s pre-CEA to 0.25 ± 0.09 m/s post-CEA (*p* < 0.05, Figure 1C).

CTA imaging in pre-CEA patients demonstrated lumen stenosis or occlusion caused by a semicircular low-density signal at the carotid bifurcation, with features such as plaque calcification, lipid core, hemorrhage, and ulceration (Figure 1D). Post-CEA, patients experienced a significant improvement in the degree of stenosis, as depicted in Figure 1E (*p* < 0.01).

CTP imaging indicated that cerebral perfusion in the ROIs on the ipsilateral side was significantly reduced compared to the contralateral side of carotid stenosis, characterized by prolonged rMTT and rTTP, as well as decreased rCBF, with no significant change in rCBV (Figure 1F). After CEA, the rMTT, rTPP, and rCBF on the lesion side in operation patients showed gradual recovery, indicating improvement in hypoperfusion (Figure 1G, *P* < 0.01).

### 6.3 Morphological and multi-modal imaging features of atherosclerotic plaque

**Figure 2:**
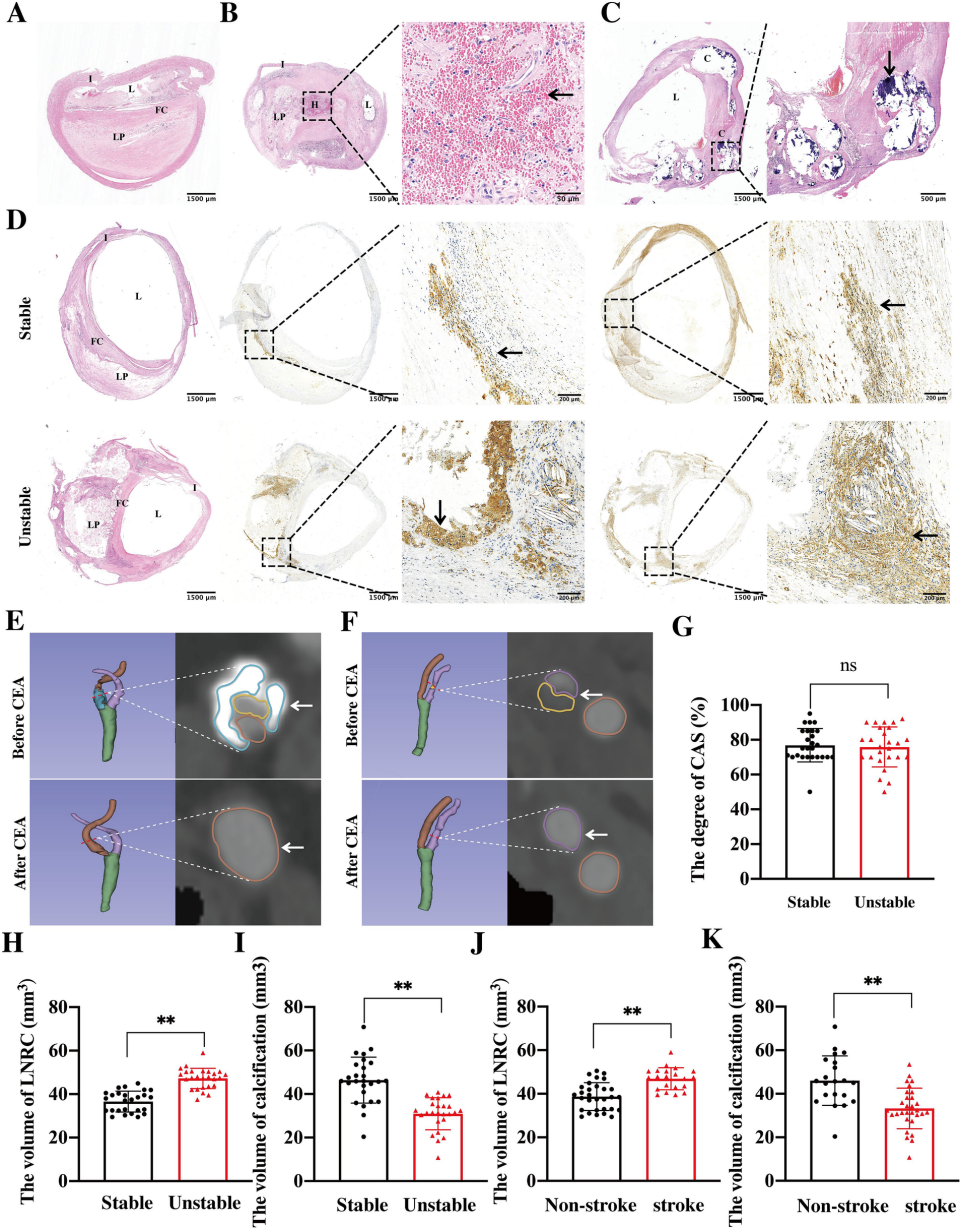
Morphological and multi-modal imaging features of atherosclerotic plaques. Carotid atherosclerotic plaques exhibited lipid cores encased by fibrous caps, which were deposited beneath the intima, resulting in severe luminal stenosis **(A)**. Additionally, intra-plaque hemorrhage (**B**, black arrow) and calcification (**C**, black arrow) were also observed within the lesions. The stable plaques appeared as small lipid cores covered by thick fibrous caps, with minimal or absent macrophage infiltration. In contrast, vulnerable plaque were characterized by LRNC covered by thin fibrous caps, with extensive macrophage infiltration **(D)**. In pre-CEA patients, both mixed (**E**, white arrow) and soft (**F**, white arrow) plaques consisting of calcification and lipid core contributed to significant luminal stenosis. And CTA visual analysis confirmed that the degree of stenosis between the stable and vulnerable plaques patients had no differences (**G**, ns indicates no statistical difference). Comparative analysis revealed that in vulnerable plaques, the volume of LRNC increased significantly (**H**, **p < 0.01), while the volume of calcification decreased significantly (**I**, **p < 0.01) when compared to stable plaques. Patients with a history of stroke or TIA before CEA also exhibited a larger volume of LRNC (**J**, **p < 0.01) and a smaller volume of calcification (**K**, **p < 0.01) when compared to those with no history of stroke or TIA before CEA. I, intima; L, lumen; FC, fibrous cap; LP, lipid core; H, hemorrhage; C, calcification.

H&E and IHC staining of CEA specimens was performed to evaluate the features of atherosclerotic plaque. H&E staining revealed eosinophilic smooth muscle fiber bundles, while IHC staining identified *α*-SMA-positive cells, indicating the plaque fibrous cap. The lipid core, observed in H&E staining, consisted of foam cells and cholesterol crystals, and IHC staining suggested the presence of CD68-positive macrophage infiltration within the plaque. As shown in Figure 2A, atherosclerotic plaque was characterized by a lipid core covered by a fibrous cap, which is deposited beneath the intima, leading to significant luminal stenosis. Intra-plaque hemorrhage (Figure 2B) and calcification (Figure 2C) were also observed in plaque. Stable plaque were characterized by small lipid cores covered by thick fibrous caps, with minimal macrophage infiltration, whereas vulnerable plaque presented with LRNC covered by a thin fibrous cap, accompanied by significant macrophage infiltration (Figure 2D).

To further quantify the size of atherosclerotic plaque, 3D Slicer software was used to reconstruct and quantify the volumes of the lipid core, calcification, and stenosis in CAS patients. The results indicated that in pre-CEA patients, both mixed plaque (Figure 2E) consisting of calcification and lipid cores, and soft plaque (Figure 2F) consisting of lipid cores were observed in the stenosis of carotid artery. Among the operation patients, 26 out of 52 patients were identified as having either stable and vulnerable plaque. However, there was no significant differences in the degree of CAS between the stable and vulnerable plaque (Figure 2G). The volume of LRNC was significantly higher (Figure 2H), whereas the volume of calcification (Figure 2I) was significantly lower in unstable plaque when compared to stable plaque. Additionally, 21 patients in the operation group had experienced stroke/TIA prior to CEA. Patients with ipsilateral symptomatic CAS exhibited a larger LRNC volume (Figure 2J) and a smaller calcification volume (Figure 2K). These findings consistent with the characteristics observed in patients with vulnerable plaque. Thus, to prevent ischemic stroke, attention should be directed not only toward stenosis but also toward plaque stability.

### 6.4 Changes in chemokines pre- and post-CEA

**Figure 3:**
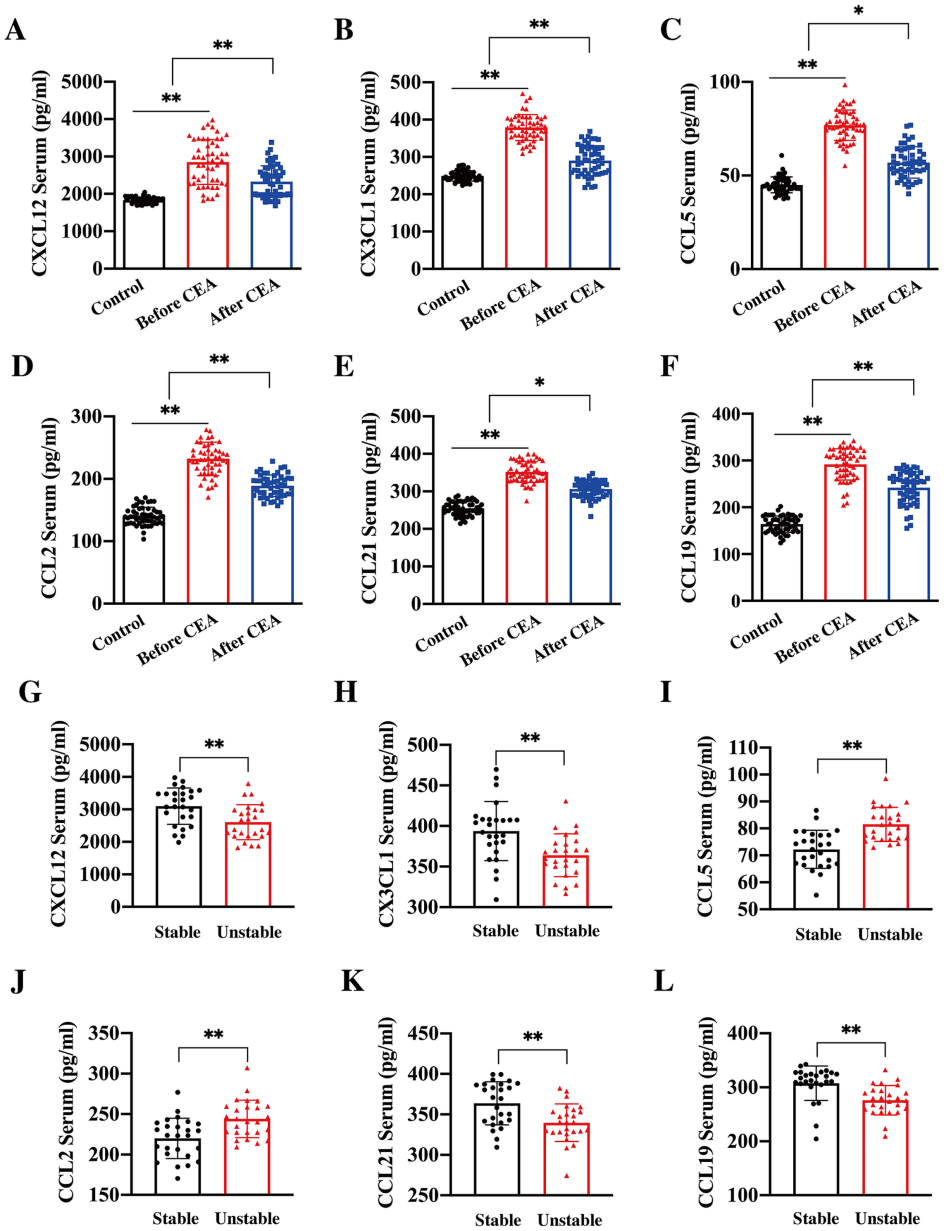
Changes in serum chemokine levels pre- and post-CEA. Serum concentrations of CXCL12 (**A**, **p < 0.01), CX3CL1 (**B**, **p < 0.01), CCL5 (**C**, **p < 0.01, *p < 0.05), CCL2 (**D**, **p < 0.01), CCL21 (**E**, **p < 0.01, *p < 0.05), and CCL19 (**F**, **p < 0.01) were significantly elevated in patients with CAS compared to controls. Notably, levels of these chemokines significantly decreased by the 7th day post-CEA. Further stratification of chemokine levels according to plaque features revealed that the concentrations of CXCL12 (**G**, **p < 0.01), CX3CL1 (**H**, **p < 0.01), CCL21 (I, **p < 0.01), and CCL19 (**J**, **p < 0.01) were significantly higher in patients with stable plaques, whereas CCL2 (**K**, **p < 0.01) and CCL5 (**L**, **p < 0.01) were significantly elevated in patients with unstable plaques.

Chemokines are known to be pivotal in the formation and progression of atherosclerosis. In this study, we further explored the relationship between serum chemokine levels and plaque stability in CAS patients, thereby assessing the impact on stroke risk. Serum concentrations of CCL2, CCL5, CXCL12, CX3CL1, CCL19, and CCL21 were significantly elevated in CAS patients compared to controls. Following CEA, the levels of these chemokines decreased significantly by the 7th day post-operation (Figures 3A–F). In addition, it was found that serum levels of CXCL12, CX3CL1, CCL21, and CCL19 were significantly higher (Figures 3G–J), while levels of CCL2 and CCL5 were lower (Figures 3K,L) in patients with stable plaque when compared to those in patients with vulnerable plaque. These findings suggest distinct biological roles for these chemokines in plaques formation and progression, influencing plaque stability and clinical outcomes.

In the operation group, 41 patients were receiving statin therapy prior to CEA, a proportion significantly higher than patients observed in the control group. However, subgroup analysis revealed that statin use had no effect on serum chemokine concentrations before CEA (*p* > 0.05). In addition, 21 patients in the operation group had experienced ischemic cerebrovascular events prior to CEA. To assess whether preoperative stroke/TIA influenced the levels of chemokines in serum, we further stratified the operation group into symptomatic and asymptomatic stenosis subgroups based on the presence of ischemic cerebrovascular events before surgery. The results showed no significant differences (*p* > 0.05) in serum chemokine levels between the ipsilateral symptomatic and asymptomatic patients within operation group. We hypothesize that this lack of difference may be attributable to the fact that all patients underwent CEA at least two weeks after the stroke/TIA events, allowing for the stabilization of their condition and minimizing the potential preoperative effects of stroke on serum chemokine levels.

### 6.5 Chemokines combined with imaging predict plaque stability

**Figure 4:**
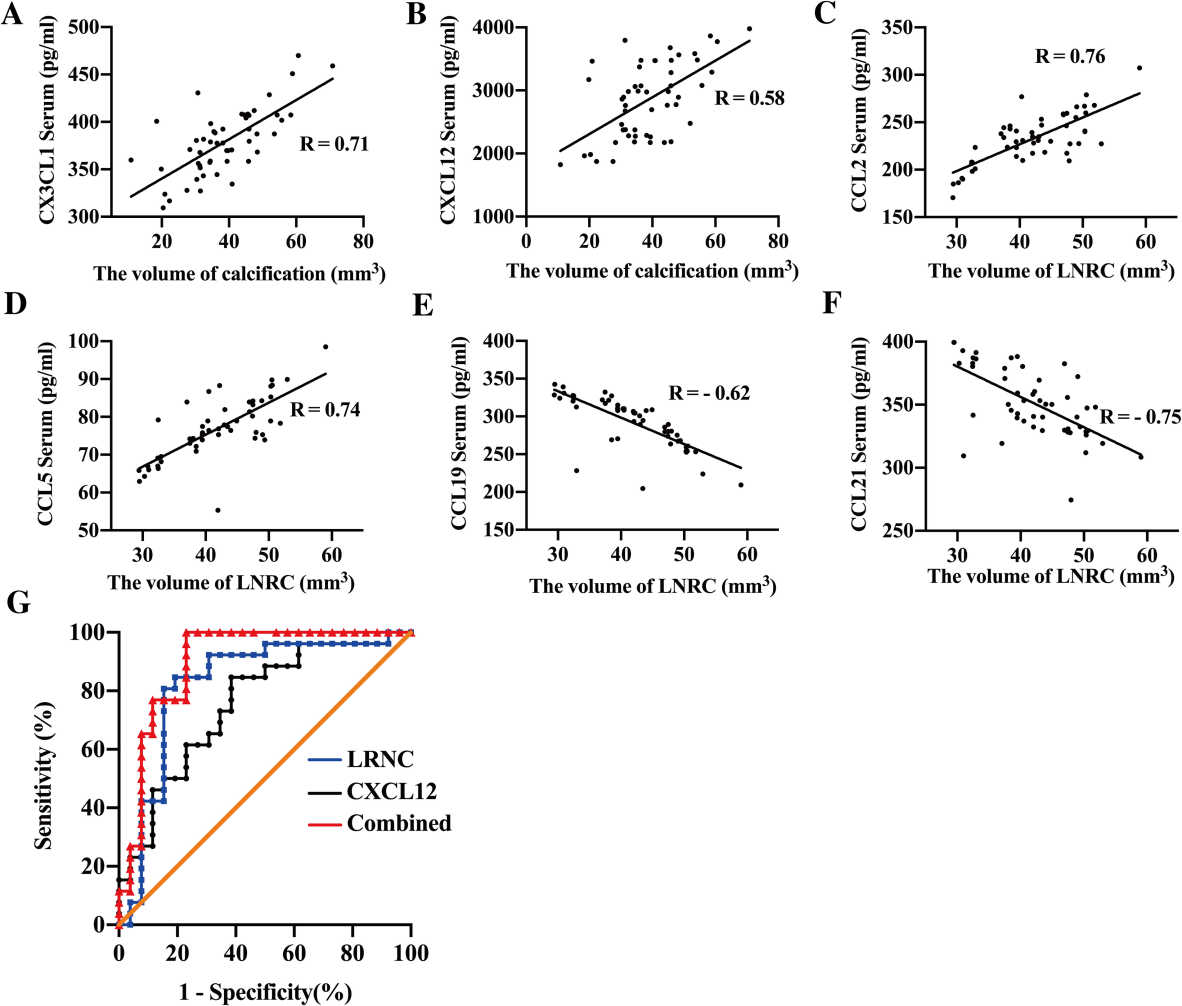
Chemokines combined with imaging predict plaque stability. Correlation analysis revealed that circulating levels of CX3CL1 (**A**, *R* = 0.71) and CXCL12 (**B**, *R* = 0.58) were positively correlated with the volume of plaque calcification in patients with carotid artery stenosis (CAS). Serum concentrations of CCL2 (**C**, *R* = 0.76) and CCL5 (**D**, *R* = 0.74) were positively associated, whereas CCL19 (**E**, *R* = −0.62) and CCL21 (**F**, *R* = −0.75) showed a negative correlation with LRNC volume. When combined, serum CXCL12 and LRNC volume demonstrated enhanced predictive ability for plaque stability (AUC = 0.9001), with sensitivity and specific of 87.31 and 92.31%, respectively **(G)**.

Correlation analysis between serum chemokines and plaque characteristics, as assessed by CTA, revealed that CX3CL1 (*p* < 0.01, *R* = 0.71) and CXCL12 (*p* < 0.01, *R* = 0.58) were positively correlated with plaque calcification volumes as shown in Figures 4A,B. Higher serum levels of CX3CL1 and CXCL12 were associated with larger calcification volumes. Furthermore, serum concentrations of CCL2 (*p* < 0.01, *R* = 0.76) and CCL5 (*p* < 0.01, *R* = 0.74) were positively correlated with LRNC volumes as shown in Figures 4C,D, while CCL19 (*p* < 0.01, *R* = − 0.62) and CCL21 (*p* < 0.01, *R* = − 0.75) were negatively correlated with LRNC volumes shown in Figures 4E,F. These findings suggested that chemokines contribute to atherosclerotic progression via distinct biological pathways. CCL2 and CCL5 are primarily involved in monocyte and SMC recruitment, promoting the formation of atherosclerosis, whereas CCL19 and CCL21 facilitate macrophage emigration, thereby promoting the regression of advanced plaques.

Subsequent univariate and multivariate logistic regression analyses were conducted to determine whether serum chemokines and CTA parameters could serve as predictors of plaque vulnerability in CAS patients. As shown in Table 2, univariate analysis identified serum levels of CCL19 (OR = 0.9634, 95% CI: 0.9382–0.9843, *p* = 0.0021), CCL21 (OR = 0.9618, 95% CI: 0.9345–0.9852, *p* = 0.0034), CX3CL1 (OR = 0.9693, 95% CI: 0.9459–0.9886, *p* = 0.005), and CXCL12 (OR = 0.9984, 95% CI: 0.9972–0.9994, *p* = 0.0046) as protective factors against plaque vulnerability. Conversely, CCL2 (OR = 1.046, 95% CI: 1.018–1.082, *p* = 0.0033) and CCL5 (OR = 1.264, 95% CI: 1.125–1.4853, *p* = 0.0007) were identified as risk factors for plaque stability. Imaging indicators, such as the volume of LRNC (OR = 1.645, 95% CI: 1.317–2.348, *p* = 0.0005), is considered as risk factor, while the volume of calcification (OR = 0.7996, 95% CI: 0.6916–0.8863, *p* = 0.0003) is regarded as a protective factor for plaque stability. However, the degree of carotid stenosis shows no correlation with plaque stability (OR = 0.9907, 95% CI: 0.9387–1.044, *p* = 0.7268). Multivariate analysis, as shown in Table 3, further indicated that serum CXCL12 (OR = 0.9977, 95% CI: 0.9956–0.9993, *p* = 0.0124) and LRNC volume (OR = 1.203, 95% CI: 1.036–1.554, *p* = 0.04) serve as independent protective and risk factors, respectively, in the prediction of plaque stability. The AUC for CXCL12 and LRNC in predicting plaque stability were 0.7544 and 0.8462, respectively. When both factors were combined, the AUC increased to 0.9001, with sensitivity and specificity of 87.31 and 92.31% respectively, as shown in Table 4 and Figure 4G.

**Table 2:** Univariate logistic regression analyses.

Variables	Univariable	
Chemokines	OR (95% CI)	*P*
CCL2	1.046 (1.018–1.082)	0.0033**
CCL5	1.264 (1.125–1.4853)	0.0007**
CCL19	0.9634 (0.9382–0.9843)	0.0021**
CCL21	0.9618 (0.9345–0.9852)	0.0034**
CX3CL1	0.9693 (0.9459–0.9886)	0.005**
CXCL12	0.9984 (0.9972–0.9994)	0.0046**
Imaging parameters		
Stenosis	0.9907 (0.9387–1.044)	0.7268
LRNC	1.645 (1.317 to 2.348)	0.0005**
Calcification	0.7996 (0.6916–0.8863)	0.0003**

**P < 0.01, indicates statistical significance.

**Table 3:** Mulvariate logistic regression analyses.

Variables	Mulvariable	
Chemokines	OR (95% CI)	*P*
CXCL12	0.9977 (0.9956–0.9993)	0.0124*
Imaging parameters		
LRNC	1.203 (1.036–1.554)	0.0410*

*P < 0.01, indicates statistical significance.

**Table 4:** Prediction of chemokines and imaging parameters for plaque stability.

Variables	AUC	95%CI	Specificity	Sensitivity	*P*
CXCL12	0.7544	0.6223–0.8866	64.29	66.67	<0.01**
LRNC	0.8462	0.7318–9,605	85.19	88.00	<0.01**
Combined	0.9001	0.8907–0.9372	92.31	87.31	<0.01**

**P < 0.01, indicates statistical significance.

## 7 Discussion

The primary mechanism underlying stroke is the embolization of thrombotic material resulting from the rupture of vulnerable atherosclerotic plaque, which subsequently obstructs distal arteries and leads to hypoperfusion in the affected regions (44). For patients with CAS, the main treatment options include stent implantation and CEA. Despite their widespread use for both symptomatic and asymptomatic patients, these procedures exhibit comparable outcomes in terms of myocardial infarction, stroke recurrence, and mortality (45). However, there remains no consensus regarding the optimal approach for the preoperative assessment of high-risk plaque, nor is there an established standardized protocol for their evaluation. To address this gap, the current study employed a multi-modal imaging approach—including CTA, CTP, carotid artery ultrasound, and angiography—to assess both the degree of stenosis and the specific morphological characteristics of plaque in patients with CAS, with the morphology of post-CEA specimens serving as the reference standard.

Our findings align with previous literature (46), indicating that 50% of the post-CEA specimens were identified as vulnerable plaque, characterized by an LRNC covered by a thin fibrous cap, accompanied by intra-plaque hemorrhage and extensive macrophage infiltration. Conversely, stable plaque displayed smaller lipid cores beneath a thick fibrous cap, with accompanying calcification and minimal or no macrophage infiltration. Notably, the degree of lumen stenosis did not differ significantly between patients with stable versus vulnerable plaque. These findings underscore the importance of combining multiple imaging modalities to facilitate accurate preoperative assessment and identification of high-risk plaque in patients undergoing CEA. A comprehensive evaluation beyond simply quantifying CAS is crucial for the effective prevention and management of ischemic stroke.

In addition to imaging-based evaluation, the assessment of lumen stenosis, identification of high-risk atherosclerotic plaque, and monitoring of postoperative complications through serological markers are also vital for patients undergoing CEA. Previous studies (17–33, 47) have consistently demonstrated elevated chemokine levels in animal models and in patients with atherosclerosis. These elevated chemokines play critical roles in the biological processes of monocyte adhesion (25), recruitment (17–20), and migration (19, 25); macrophage proliferation (21–24), phagocytosis (19, 20), apoptosis (29), and egress (32–34); as well as smooth muscle cell (SMC) recruitment (31) and osteogenic transformation (30). Additionally, sequence variations in the promoter regions of CCL19 and CCL21 have been associated with an increased susceptibility to myocardial infarction (48), while genetic predisposition to elevated circulating levels of CCL2 correlates with a heightened risk of stroke (49).

In the present study, we observed that patients with CAS had significantly elevated serum levels of CX3CL1, CXCL12, CCL19, CCL21, CCL2, and CCL5 compared to control individuals, suggesting that these chemokines are intricately involved in the initiation and progression of atherosclerosis. Notably, by the 7th day post-CEA, serum levels of these chemokines had significantly decreased, implying that removal of atherosclerotic plaque via CEA reduces systemic chemokine expression. Furthermore, we found that patients with vulnerable plaque had higher concentrations of CCL2 and CCL5 and lower levels of CX3CL1, CXCL12, CCL19, and CCL21 compared to those with stable plaque. Correlation analysis revealed that CX3CL1 and CXCL12 levels were positively associated with plaque calcification volume. Calcification is typically associated with more stable plaque and a lower risk of rupture, whereas CCL2 and CCL5 levels were positively correlated, and CCL19 and CCL21 levels negatively correlated, with the volume of lipid core within plaque.

These findings suggest that the differential size of the lipid core in plaque is influenced by the distinct biological functions of these chemokines. Specifically, CCL2 and CCL5 are involved in the recruitment and aggregation of monocytes, promoting their migration into plaque and contributing to plaque progression (19–24). Conversely, CCL19 and CCL21 promote the egress of macrophages from within plaque, thus facilitating plaque regression (32–34, 50). Collectively, these data indicate that CCL2 and CCL5 promote lipid core enlargement and plaque progression, whereas CCL19 and CCL21 contribute to the regression of advanced plaque. Our multivariate logistic regression analysis further indicated that CXCL12 is an independent protective factor for plaque stability, a finding consistent with the work of Akhtar et al. (31), who reported more stable plaque and reduced atherosclerotic burden in mice injected with CXCL12 (51).

## 8 Conclusion

Our findings support the integration of multi-modal imaging techniques with serological markers to improve the identification of high-risk atherosclerotic plaque in patients with CAS. Such an approach enables more accurate preoperative assessment, better prediction of stroke risk, and enhanced monitoring of postoperative prognosis.

### 8.1 Limitations

This study has several limitations. First, the sample size was relatively small due to the constraints inherent in clinical trials, which may limit the generalizability of our findings. Second, the follow-up period was short, only extending from pre-CEA to 7 days post-surgery. Longer follow-up durations, ideally one year or more, could provide more comprehensive data regarding stroke prevention and recurrence rates following CEA. Lastly, due to practical considerations for hospitalized patients, this study employed CTA for plaque stability assessment and CTP for cerebral perfusion evaluation, but lacked MRA to provide a more detailed characterization of plaque features. Future studies incorporating MRA could yield additional insights into plaque composition and vulnerability.

## Data Availability

The raw data supporting the conclusions of this article will be made available by the authors, without undue reservation.
